# Cohort profile: The Corona Behavioral Unit cohort, a longitudinal mixed-methods study on COVID-19-related behavior, well-being and policy support in the Netherlands

**DOI:** 10.1371/journal.pone.0289294

**Published:** 2023-07-31

**Authors:** Wijnand van den Boom, Mart van Dijk, Bianca Snijders, Guus Luijben, Jan van der Laan, Saskia Euser, Jet G. Sanders, Anne H. Buitenhuis, Pita Spruijt, Floor Kroese, Mattijs Lambooij, Yvette Muhren, Nannah Tak, Koen van der Swaluw, Caroline van Rossum, Thijs Nielen, Janneke Elberse, Reint Jan Renes, Mariken Leurs, Marijn de Bruin

**Affiliations:** 1 National Institute for Public Health and the Environment (RIVM), Bilthoven, The Netherlands; 2 University of Utrecht, Utrecht, The Netherlands; 3 Erasmus School of Health Policy and Management, Erasmus University, Rotterdam, The Netherlands; 4 GGD GHOR Nederland, Utrecht, The Netherlands; 5 Radboud University, Nijmegen, The Netherlands; 6 Amsterdam University of Applied Sciences, Amsterdam, The Netherlands; 7 Radboud University Medical Center, Institute of Health Sciences, IQ Healthcare, Nijmegen, The Netherlands; Regional Health Care and Social Agency of Lodi, ITALY

## Abstract

This ‘cohort profile’ aims to provide a description of the study design, methodology, and baseline characteristics of the participants in the Corona Behavioral Unit cohort. This cohort was established in response to the COVID-19 pandemic by the Dutch National Institute for Public Health and the Environment (RIVM) and the regional public health services. The aim was to investigate adherence of and support for COVID-19 prevention measures, psychosocial determinants of COVID-19 behaviors, well-being, COVID-19 vaccination, and media use. The cohort also examined specific motivations and beliefs, such as for vaccination, which were collected through either closed-ended items or open text responses. In April 2020, 89,943 participants aged 16 years and older were recruited from existing nation-wide panels. Between May 2020 and September 2022, 99,676 additional participants were recruited through online social media platforms and mailing lists of higher education organizations. Participants who consented were initially invited every three weeks (5 rounds), then every six weeks (13 rounds), and since the summer of 2022 every 12 weeks (3 rounds). To date, 66% of participants were female, 30% were 39 years and younger, and 54% completed two or more questionnaires, with an average of 9.2 (SD = 5.7) questionnaires. The Corona Behavioral Unit COVID-19 cohort has published detailed insights into longitudinal patterns of COVID-19 related behaviors, support of COVID-19 preventive measures, as well as peoples’ mental wellbeing in relation to the stringency of these measures. The results have informed COVID-19 policy making and pandemic communication in the Netherlands throughout the COVID-19 pandemic. The cohort data will continuously be used to examine COVID-19 related outcomes for scientific analyses, as well as to inform future pandemic preparedness plans.

## Introduction

Behavior plays a significant role in reducing the spread of SARS-CoV-2, the virus that causes the disease known as COVID-19 [1]. During the COVID-19 pandemic, governments around the world, including the Dutch government, have placed nonpharmaceutical interventions to reduce the spread of COVID-19, from recommendations on sanitization and social distancing to regulation on mask wearing and nation-wide lockdowns [2]. These COVID-19 preventive measures were designed to limit the number of social contacts and make social encounters less likely to result in transmission of the virus. Therefore, understanding the public’s adherence to these measures and the impact they have on behaviors (eg, social activities), its psychosocial determinants (eg, risk perceptions, self-efficacy or trust in government) and related well-being, is key [3]. Such information can inform the directionality of national and local policy and communication decisions, and aid the effectiveness of the (future) pandemic responses.

In March 2020, during the early stages of the COVID-19 pandemic in the Netherlands, the Corona Behavioral Unit (CBU) was established by the Dutch National Institute of Public Health and the Environment (RIVM). In collaboration with the 25 regional Public Health Services (GGDs), in April 2020 the CBU launched a longitudinal cohort study using an online questionnaire to monitor behavior, its determinants, the support base for COVID-19 preventive measures, and well-being over time. We opted for a cohort rather than representative cross-sectional polls, as this would allow us to conduct longitudinal explanatory analyses. Also, in-depth qualitative research was integrated in the design using open-ended questions, and after finishing the questionnaire participants were invited to be interviewed or to participate in focus groups.

Findings from the cohort study have informed national as well as regional-level COVID-19 policies, communication strategy and campaigns. Results have also been used by the media to inform citizens of developments during the pandemic. The objectives of this Cohort Profile are to describe the cohort’s design, recruitment, data collection (including an overview of some of the key questionnaire items), baseline participant characteristics, key published findings to date, and plans for future research and publications.

## Cohort description

### Design

The study adopted a nationwide longitudinal dynamic cohort approach, of both quantitative research methods (online questionnaire including closed- and open-ended questions) and qualitative research methods (telephone interviews, online focus groups). The frequency of the online questionnaire was every three weeks (data collection April–June 2020; rounds 1 to 4), every six weeks (August 2020 –March 2022; rounds 5 to 19), and between rounds 19 and 21 (March–June 2022) there was an interval of 13 weeks.

### Recruitment and data collection

In April 2020, individuals aged 16 years and older who participated in one of 25 regional public health service (GGD) panels (GGD Amsterdam, GGD Brabant-Zuidoost, GGD Drenthe, GGD Flevoland, GGD Fryslân, GGD Gelderland-Midden, GGD Gelderland-Zuid, GGD Gooi en Vechtstreek, GGD Groningen, GGD Haaglanden, GGD Hart voor Brabant, GGD Hollands Midden, GGD Hollands Noorden, GGD IJsselland, GGD Kennemerland, GGD Limburg-Noord, GGD Noord- en Oost-Gelderland, GGD Regio Utrecht, GGD Rotterdam-Rijnmond, GGD Twente, GGD West-Brabant, GGD Zaanstreek-Waterland, GGD Zeeland, GGD Zuid-Holland Zuid, GGD Zuid Limburg) and eight municipal panels (Den Haag, Delft, Westland, Schiedam, Capelle a/d IJssel, Vlaardingen, Maasluis, Nissewaard) were invited to participate in a questionnaire on behavior and subjective well-being during the corona pandemic. These panels each consist of 1,000 to 10,000 participants who are invited to fill in questionnaires about health-related topics or other topics a few times each year. Participants who completed the questionnaire were asked if they were willing to receive invitations for future questionnaires. As we anticipated dropout over time, we decided to recruit additional participants every other round of data collection. Therefore, a link (referred to as ‘open link’) was shared on social media channels (eg, Facebook) and through mailing lists of higher education organizations such as The Dutch National Youth Council (NJR) and MBO Council (MBO Raad) to recruit as many people as possible. Due to holiday periods, availability of staff and other practicalities (eg, timing of the Dutch public health monitor), this took only place during rounds 3, 5, 6, 8, 10, 12, 13, 15, 17 and 19. There was a focus on recruiting those people who were underrepresented in the cohort, particularly individuals aged 16 to 24 years.

### Procedures and informed consent

All participants 16 years and older provided online written informed consent before filling out each questionnaire, in compliance with the Dutch Law for Research Involving Human Subjects (WMO; see S1 Text). Respondents who completed the questionnaire for the first time were asked whether they were willing to participate in future questionnaires, and to participate in in-depth interviews or focus groups. If yes, these participants were asked to provide their email address so that they could receive an invitation containing a unique link, which allowed tracking of the participant throughout the cohort study. Participants could opt out by using an opt-out link in the invitation and were also informed that they could opt out at any time by sending an email to the research agent. Participants could also indicate to have their data deleted from the dataset if they wished so.

### Measures

#### Quantitative data: Online questionnaire

**Fig 1:**
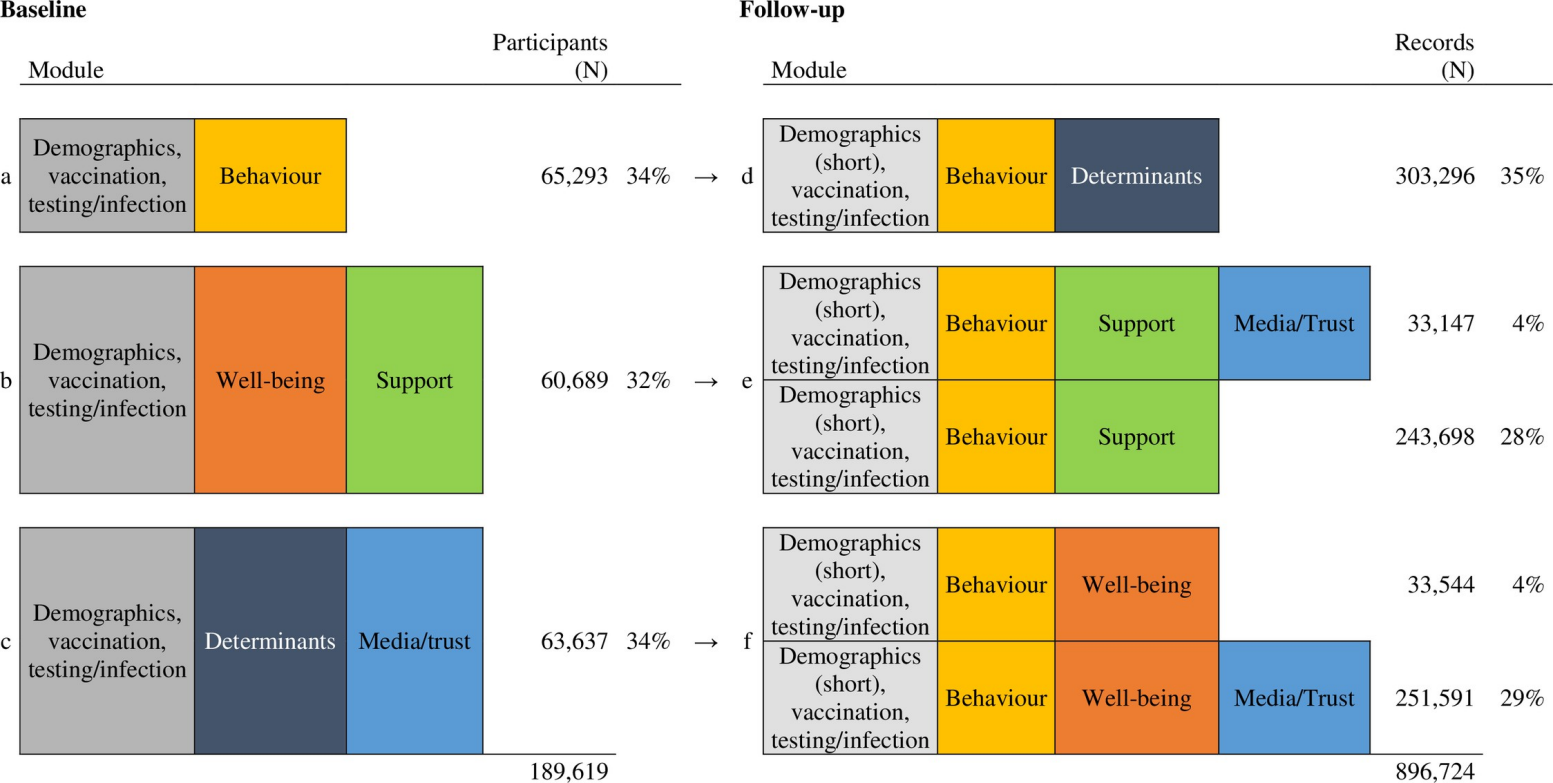
Questionnaire modules in baseline and follow up questionnaires, Corona Behavioral Unit cohort study, April 2020 –September 2022. In follow-up questionnaires, all participants filled out a short version of the sociodemographic module with items that could potentially have changed over time (eg, medical health condition), that were needed for linkage purposes (i.e., age and sex–see more details below), were included for routing purposes (eg, having kids for questions related to kids), or were related to COVID-19 vaccination, testing and infection. Also, all participants filled out questions from the behavior module, and, based on the baseline module and random allocation during follow-up, was complemented with questions from a module on either (d) psychosocial determinants of adherence, (e) policy support, use of media and trust in the COVID-19 approach of the Dutch government, or (f) well-being, use of media and trust in the COVID-19 approach of the Dutch government (Fig 1).

At baseline, all participants completed an extensive section on demographics (eg, age, educational level, gender, country of birth, living and work situation), socioeconomic status (eg, unemployment, change in financial situation due to the COVID-19 pandemic), health status (eg, having a pre-existing physical medical health condition), and questions on COVID-19 vaccination, testing and infection. Then, they were randomly assigned to the following (combination of) modules (Fig 1): (a) behavior (adherence and social activity), (b) well-being and policy support, and (c) psychosocial determinants of adherence to COVID-19 preventive measures, use of media and trust in the COVID-19 approach of the Dutch government. We opted for this approach to reduce the questionnaire’s length to minimize attrition.

Where possible, existing validated scales or items were used (eg, to assess background variables and well-being). The specific and novel context of the COVID-19 pandemic also demanded developing new items (eg, to assess adherence to and support for COVID-19 preventive measures). This was done by senior researchers from the Corona Behavioral Unit and by at least two external senior researchers from the academic advisory board with expertise in both the specific area and survey design. Every module was introduced to the participant and specific care was taken to formulate questions in a way to minimalize socially desirable answers (eg, by legitimizing non-adherence to the measures) and prompt reliable responses (eg, by asking about very specific behaviors within clearly defined time periods).

A selection of the measures as used in our questionnaires are presented in Table 1. Items in the behavior module related to adherence to COVID-19 preventive measures and social activity. For example, adherence to social distancing in different settings such as when doing groceries or when at work, and testing when symptomatic or isolating after a positive coronavirus test result. This module also included a series of questions on social activity (eg, receiving visitors in the previous week) and mobility (eg, leaving the house to go to work). Items in the vaccination, testing and infection module included COVID-19 vaccination (eg, intention to get vaccinated, reasons for not getting the vaccine), COVID-19 symptoms, (self-) testing for a corona virus infection, having had a (recent) corona virus infection, and adherence to the quarantine and isolation measures.

**Table 1:** Main measures in baseline and follow up questionnaires of the Corona Behavioral Unit cohort study, April 2020 –September 2022.

Module	Topics
Demographics (baseline)	Sex, age, municipality, education, country of birth, vulnerable health, living conditions, work, financial situation, 1.5m distancing in work situation, persistent complaints after COVID-19 infection.
Demographics (follow-up)	Sex, age, vulnerable health, living conditions, work, 1.5m distancing in work situation, work, financial situation, persistent complaints after COVID-19 infection.
Vaccination, testing, infection	• COVID-19 vaccination, symptoms, (self-)testing, infection, adherence to quarantine and isolation measures.
Behavior	• Adherence to COVID-19 preventive measures: washing hands, use of paper towel, sneezing in elbow, keeping distance (in certain situations), not shaking hands, wearing face masks, working from home, ventilating the house. • Social activity and mobility: number of times leaving the house in different situations, having visitors at home, visiting others, going on holidays, visiting high risk countries.
Wellbeing	• Self-perceived general health, satisfaction with life, mental health, loneliness, quality of social contacts, coping with COVID-19 situation, COVID-19 pandemic fatigue, experiences positive and negative effects. • Lifestyle & health: exercise, dietary patterns, alcohol use, smoking.
Trust and Media use	• Trust in the COVID-19 approach of the Dutch government, comparing the Dutch approach to other countries, conversations with others about the approach. • Following the news on COVID-19, important source of information about the corona virus. • Procedural and distributive justice.
Policy Support	• Policy support for current (at time of data collection) and future COVID-19 measures as taken by the Dutch government. • Procedural and distributive justice (i.e., how people feel they’re being treated, and whether they experience the Dutch government’s measures as being fair).
Determinants of adherence	• COVID-19 risk perception (i.e., estimation of chance and seriousness of getting infected yourself or infecting others; concerns about a new corona virus variant). • Emotional response (i.e., the perception of how fast the virus is spreading and how much worry, stress, fear people experience). • Self-efficacy (i.e., the participant’s self-appraisal of their own competencies to comply to specific COVID-19 preventive measures). • Response efficacy (i.e., the perceived effectiveness of adherence to the COVID-19 preventive measures for reducing the spread of the virus). • Descriptive norms (i.e., the perception of which specific COVID-19 preventive measures are typically performed by significant others).

Note. From round 15 onwards, items on procedural justice are included in the Policy Support module, in addition to the Trust and Media use module.

Items in the well-being module related to mental health (Mental Health Inventory-5 [4]), life satisfaction [5], loneliness (6-item De Jong-Gierveld Loneliness Scale [6]), resilience and quality of social contacts [6–8]. In this module we also assessed participants’ lifestyle: physical exercise, dietary patterns, alcohol use and smoking. The module trust and media use included questions related to trust in the COVID-19 prevention approach by the Dutch government, and questions on use of media in gathering (online) information on COVID-19.

Items in the policy support module related to support for current and future COVID-19 preventive measures as taken by the Dutch government to control the spread of the coronavirus in the Netherlands. Items in the psychosocial determinants of adherence module related to risk perception, emotional response to the virus (eg, perceived threat [9]), response efficacy (participants perception of the effectiveness of prevention behaviors and policies), self-efficacy (confidence in one’s ability to perform the behavior), and descriptive norms (the perception about the behavior of others), in relation to the current COVID-19 preventive measures. Items in these two modules focused only on those measures that involved participants to make choices about their behavior (eg, sneezing in the elbow and coronavirus self-testing), but not measures for which their own behavior had no influence, such as closures of restaurants or schools.

Items in all modules were adjusted, removed, or added over the rounds to serve as input for new policy as well as to align with it. Adjustments to items were based on COVID-19 preventive measures that were imposed by the Dutch government at the time of data collection or based on respondents’ answers to open-ended questions. Adjustments could also been based on information retrieved from our open-ended questions or interviews during which respondents mentioned relevant topics or aspects that we had not yet included, or when the scientific board or project members identified new and upcoming questions when interacting with policymakers. Questionnaires (in Dutch) are available at https://nationaalgeoregister.nl/geonetwork/srv/dut/catalog.search#/metadata/3639da42-78b4-466c-a3eb-9e67809405d2.

#### Open-ended questions

Open-ended questions were embedded in the online questionnaire to obtain an understanding of participants’ beliefs, experiences or concerns across a range of behaviors throughout the rounds. Examples include reasons (not) to participate in testing, tracing and isolating, (not) getting a COVID-19 vaccine, or experienced positive and negative effects of the pandemic and its policies on well-being. At the end of each questionnaire an open-ended question was included where participants could indicate whether they missed any questions related to COVID-19 (round 1–7), had remarks related to the questionnaire (from round 7 onwards) and whether they had any additional thoughts in relation to the COVID-19 preventive measures in general (from round 7 onwards).

Responses were coded using a thematic codebook. For every question a fitting codebook was developed, based on theoretical foundations of the (or most closely relevant) behavior identified from the literature and aligned with previous questionnaire and interview findings (top-down). This was supplemented by themes mentioned in the response set (bottom-up). To test the codebooks and identify new codes, a sample of responses was coded by two independent primary coders. Findings were discussed, and the codebook was refined. A team of secondary coders (between two and ten) then used the codebook and protocol to code the rest of the data. Following instructions, each secondary coder coded 100–200 responses to become acquainted with the data. They discussed cases of doubt with one of the primary coders, after which the codebook was updated, and decisions were logged. Cases of doubt were discussed within the coding team throughout the coding process. In case of a large number of responses, coders were asked whether shifts in key themes still occurred or new themes came up (saturation check). After coding, the codebook underwent further refining and accuracy checks were performed against the final codebook and decision log by one of the primary coders. Codes were integrated to identify main themes for each question. If new codes or themes were identified, in case of longitudinal questions at the end of the questionnaire, earlier data sets were recoded to allow for comparison across time. Additionally, answers could be linked to the corresponding participant number, allowing for mixed-method approaches (eg, identifying differences between groups based on demographics). Themes were used to inform interview topic guides, survey design (eg, to identify potentially relevant reasons for COVID-19 vaccination intention from the open text responses [10]), or to help understand a topic or impact of a policy change directly.

#### Qualitative data: Interviews and focus groups

The cohort study was also designed to involve qualitative interviews after each round to explore specific themes more in-depth, such as gaining insight into people’s reasoning and motives for their behavior, the impact of COVID-19 preventive measures on people’s well-being or opinions about pandemic policies. Depending on the research questions we decided to hold either interviews or focus groups. Those cohort participants who agreed after completing their first questionnaire to be contacted for interviews by phone were potentially eligible. We then examined, based on participants’ completed questionnaire, who was eligible and most informative to interview, for example based on their age or on the distribution in responses on opinions about COVID-19 vaccination (depending on the research question at the time of data collection). After the selection, email addresses and phone numbers of the selected participants were requested at the research agency. Email addresses were used to inform people that they could expect a phone call from the interview team at the RIVM.

Interviews were conducted by multiple interviewers using a standardized topic guide, recorded and were then sent to an external company for transcription. Two types of qualitative analysis are being used, 1) analyses to inform COVID-19 reports, policy makers and to improve the survey, and 2) analyses for scientific publications. For the first type of analysis, responses were added into a predetermined matrix with all topic guide themes and questions on the x-axis and each interviewee on the y-axis. Responses were added directly after each interview by interviewers, and were kept as literal quotes as much as possible. Thematic analysis was performed on this matrix by multiple researchers. Two analyses took place in parallel: one entailed getting a general overview, while the other had the aim to look at each theme separately. Both analyses were performed by at least two researchers simultaneously. After the parallel analyses (general and per theme), all researchers discussed their findings in a group session. This procedure made it possible to complete analyses and present the results within a few days after the last interview was conducted. Results derived from the thematic analyses of the interviews were used to identify perceptions and beliefs to generate response options for new survey questions. For example, interviews were used to identify concerns and beliefs surrounding COVID-19 vaccination, and these items in turn have been reported on in several policy reports and a scientific paper [10].

Regarding analyses for scientific papers based on the interview data, transcripts were analyzed through inductive thematic analysis using MAXQDA 2018 [11]. Three steps of coding–open, axial and selective–were used. In order to do so, an initial codebook was constructed by two researchers. This codebook was based on theoretical concepts from the Health Belief Model [12]. Additional codes were then added by multiple researchers during the coding process whenever the initial coding book proved insufficiently extensive. During the coding process, interrater reliability checks were performed. Once all the interviews were coded axially, multiple researchers discussed all codes to construct a final thematic coding book. Regarding analyses for future scientific papers, this may involve additional coding depending on the specific research questions and theoretical frameworks being used.

## Linkage questionnaires

Stepwise deterministic linking for the quantitative data, using participant id number, age (divided into 7 categories) and sex (female, male, other) from the baseline questionnaires, was performed to match participants on different records across the data collection rounds. In the case that participants’ responses to these questions at their follow-up questionnaire matched those at their baseline questionnaire, or in the case that participant number and sex were identical, and age was one category higher, consecutive records were considered to be of the same individual and thus were kept in the data set.

## Statistical analyses

In this Cohort Profile, we have used descriptive statistics to summarize the baseline characteristics of the study participants, overall and according to method of recruitment, whether participants were lost to follow-up (defined as not participated for at least four rounds), and whether participants subscribed for receiving follow-up questionnaires. For comparisons between groups, Pearson’s chi-square tests were performed. Analyses were performed using *Stata* [13].

## Patient and public involvement

Participants or the public were not invited to contribute to the design, recruitment, reporting and dissemination of this research.

## Ethics statement

The cohort study does not meet the requirement as laid down in the Dutch Law for Research Involving Human Subjects (WMO) and was therefore exempted by the Centre for Clinical Expertise at RIVM from formal ethical review (Study number G&M-561). Written informed consent was provided by all participants in each data collection round. Data collection was outsourced to a research agency (‘Research 2Evolve’), as well as management of email addresses and phone numbers, for the duration of the project.

## Recruitment

**Fig 2:**
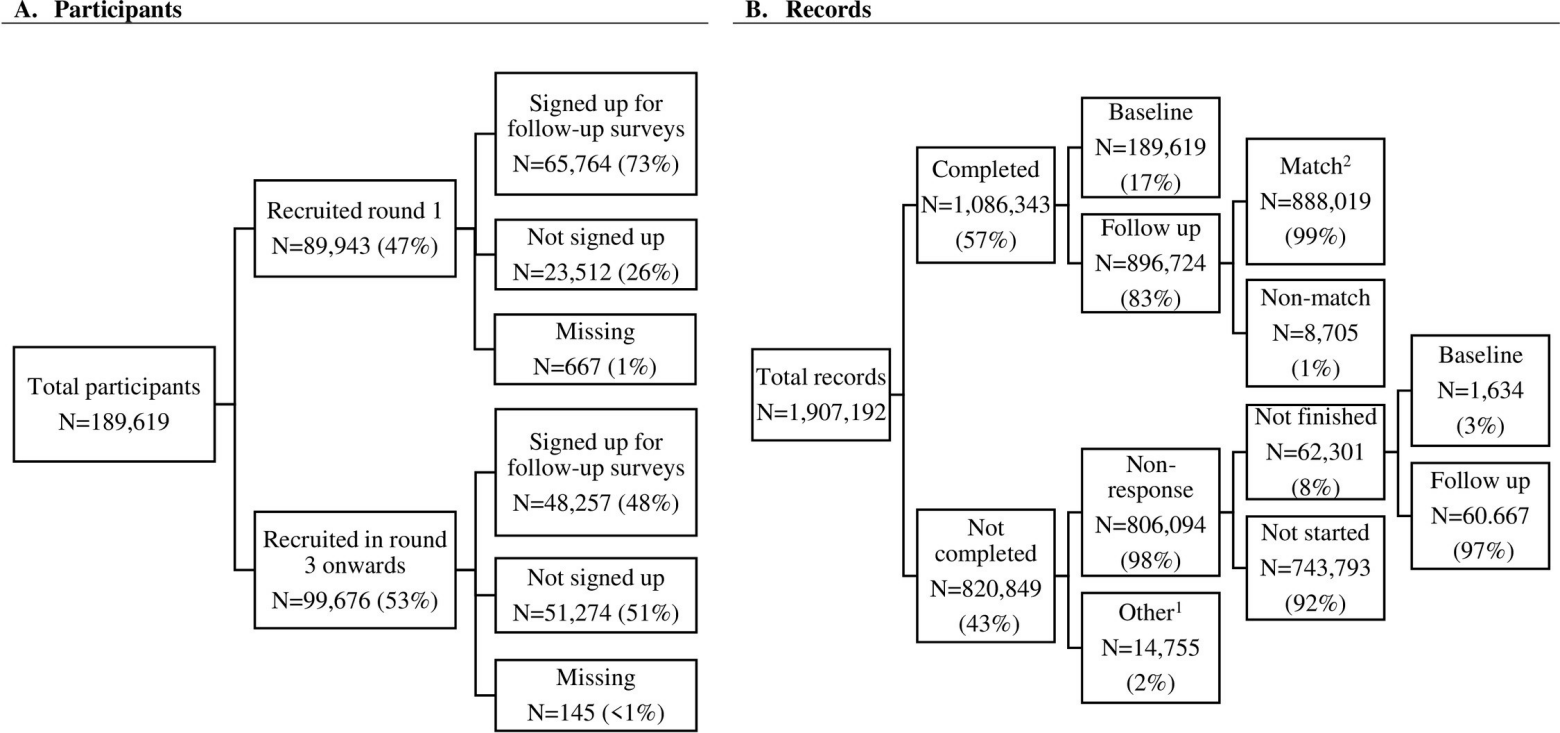
Flow diagram Corona Behavioral Unit cohort study, April 2020 –September 2022. ^1^ Other reasons for uncompleted questionnaires are: open link had been used more than once, email address was not correct, survey was closed because participant’s age was below the minimum, participant did not give permission to use their data. ^2^ Participant id number, age and sex from the baseline questionnaires were used to match participants on their follow-up questionnaires.

Between April 2020 and September 2022 (21 rounds of data collection), in total 189,619 individuals 16 years and older were recruited, 47% (N = 89,943) in round 1 via the existing Public Health Services (GGD) and municipal panels, and by sharing an ‘open link’ on social media channels such as Instagram, and 53% (N = 99,676) via such an open link from round 3 onwards (Fig 2A). An email address for follow-up was provided by 73% of participants recruited in round 1 and by 48% of participants recruited in round 3 and later.

In total, 57% of the questionnaires were completed, and 43% were not completed (Fig 2B). Of those, 98% was regarded as ‘non-response’: consisting of either participants who did not finish the questionnaire (8%; both baseline and follow-up questionnaires), or participants who did not start filling out the questionnaire after being invited (92%; follow-up questionnaires only). Other reasons for not completing were that the participant’s email address was not valid, the participant did not give permission to use their data, or that the participant did not meet the age requirements. N = 16,338 participants unsubscribed between rounds by either, a) using a link to the questionnaire that was emailed to them (first invitation or reminder), or by b) sending an email to the research agency. They were then not invited for the next round. The response rate for follow-up questionnaires was 82% in round 2 and 35% in round 20. See S1 Table for the detailed response numbers per round of data collection.

## Profile of all cohort participants

Demographic characteristics of all cohort participants are shown in Table 2. In total, 54% (N = 101,938) of participants responded to two or more questionnaires. Of those participants, the average number of completed questionnaire was 9.49 (SD = 5.95). Two thirds of the participants were female (66%), 13% was older than 70 years of age, and 6% was 24 years old or younger. More than half completed postsecondary education (college, university, professional, vocational, and technical; 56%), 84% was living with someone and 25% had a physical medical condition, such as heart disease, that could increase the likelihood of severe COVID-19 when infected (referred to as an ‘underlying medical condition’).

**Table 2:** Baseline characteristics of participants in the Corona Behavioral Unit cohort study, April 2020 –September 2022.

Characteristics	Total	Round 1 enrolment (N = 89,943)	Round 3 and later^a^ enrolment (N = 99,676)	*P*-value^b^	Retained in the study (N = 65,996)	Lost to follow up^c^ (N = 123,623)	*P*-value	Subscribed for follow-up (N = 114,021)	Not subscribed (N = 74,786)	*P*-value
**Sex**				<0.001			<0.001			<0.001
Male	63739 (34%)	34096 (53%)	29643 (47%)		23654 (37%)	40085 (63%)		39701 (62%)	23844 (38%)	
Female	125447 (66%)	55748 (44%)	69699 (56%)		43659 (35%)	81788 (65%)		74150 (59%)	50683 (41%)	
**Age**				<0.001			<0.001			<0.001
70+ years	25078 (13%)	15143 (60%)	9935 (40%)		10847 (43%)	14231 (57%)		16988 (68%)	7947 (32%)	
55-69y	52567 (28%)	27448 (52%)	25119 (48%)		23495 (45%)	29072 (55%)		34579 (66%)	17730 (34%)	
40-54y	55282 (29%)	27399 (50%)	27883 (50%)		19531 (35%)	35751 (65%)		33574 (61%)	21457 (39%)	
25-39y	46161 (24%)	17228 (37%)	28933 (63%)		11892 (26%)	34269 (74%)		24485 (53%)	21548 (47%)	
16-24y	10531 (6%)	2725 (26%)	7806 (74%)		1627 (15%)	8904 (85%)		4395 (42%)	6104 (58%)	
**Educational level** ^d^				<0.001			<0.001			<0.001
Lower	24379 (13%)	12085 (50%)	12294 (50%)		7269 (30%)	17110 (71%)		13541 (56%)	10642 (44%)	
Middle	57565 (31%)	25621 (45%)	31944 (55%)		18313 (32%)	39252 (68%)		32941 (57%)	42842 (43%)	
Higher	105413 (56%)	51283 (49%)	54130 (51%)		41031 (39%)	64382 (61%)		66217 (63%)	72842 (37%)	
**Living alone**				<0.001			<0.001			0.039
Yes	29688 (16%)	75581 (47%)	84350 (53%)		56083 (35%)	103848 (65%)		96016 (60%)	63240 (40%)	
No	159931 (84%)	14362 (48%)	15326 (52%)		11309 (38%)	18379 (62%)		18005 (61%)	11546 (39%)	
**Underlying medical condition** ^e^				0.681			<0.001			<0.001
Yes	142080 (75%)	67373 (47%)	74707 (53%)		48620 (34%)	93460 (66%)		34475 (33%)	102571 (79%)	
No	47233 (25%)	22346 (47%)	24887 (53%)		18680 (40%)	28553 (60%)		2450 (4%)	32730 (76%)	

Note that totals might not add up due to missing values.

^a^ Additional participants that were recruited every other data collection round since round 3.

^b^ Pearson chi square test.

^c^ Not participated for at least four rounds.

^d^ Lower education level represents elementary school or less; Middle education level represents secondary education (academic, vocational, and technical education); Higher education level represents postsecondary education (college, university, professional, vocational, and technical).

^e^ Physical medical condition that could increase the likelihood of severe COVID-19 when infected.

Comparisons between baseline characteristics for different groups in the study are also shown in Table 2. Compared to those who were recruited in round 1, participants who entered the cohort at later time points were more likely to be female, younger, to have completed at least academic, vocational, and technical education, and less likely to not be living alone. Note that the recruitment specifically targeted younger people and those with a lower socioeconomic status, as these groups were underrepresented in the cohort. Compared to those who were retained in the cohort, participants lost to follow up were more likely to be female, younger, and less likely to have middle or higher educational level, to be living alone or to have an underlying medical condition. Compared to those who subscribed for future participation (60%), those who did not (40%) were more likely to be female, younger, and less likely to have middle or higher educational level, to be living alone or to have an underlying medical condition. Among those who have signed up for follow-up questionnaires (N = 114,021), 24% (N = 27,879) have participated in all rounds after they enrolled into the study.

## Profile of participants who filled out open-ended questions

From round 7 onwards, between 16% (n = 5,349; round 20) to 39% (n = 17,922; round 17) of participants responded to the open-ended question asking about whether they had any additional thoughts on the imposed COVID-19 preventive measures in general. This was completed by a selective sample of participants, as illustrated by an analysis in round 20: compared to those who did not answer this question (n = 27,565), respondents who did (n = 5,273) were more likely to be female (66% vs. 61%; *p*<0.001), to have an underlying medical condition (28% vs. 25%; *p*<0.001), to have not been vaccinated (89% vs. 97%; *p*<0.001), to have a higher educational level (68% vs. 61%; *p*<0.001), and less likely to be 70+ years old (24% vs. 27%; *p* = 0.001). In each round, between 6% (n = 3,080; round 13) to 25% (n = 15,892; round 3) of participants answered the open question about whether they had remarks related to the questionnaire (no regression analyses were performed to compare groups). See S2 Table for an overview of topics of the open-ended questions that have been included.

## Profile of participants who agreed to be interviewed

At their baseline questionnaire, 21% (N = 36,925) of participants responded positively to the question if they were willing to engage in in-depth interviews or focus groups. Compared to those who were not willing to do so (n = 135,505), those who agreed to participate in interviews or focus groups were more likely to be male (39% vs. 32%); *p*<0.001), to be 40 years or older (77% vs. 68%; *p*<0.001), to have a higher educational level (65% vs. 54%; *p*<0.001), to live with someone (85% vs. 83%; *p*<0.001), and to not have an underlying medical condition (28% vs. 24%; *p*<0.001).

Because of the large group of participants that was willing to participate in interviews, we were able to invite those that were eligible and most informative based on the research question at hand at time of data collection (eg, participants who were not willing to vaccinate). Each round, between 16 and 106 participants were selected and invited. The response rate ranged from 57% to 97%. Participants were typically invited once, except for 17 participants who were invited to be part of a qualitative interview cohort group. Because two of them dropped out, two extra participants were recruited. Of those 19 participants, 12 participated 12 times.

## Findings to date

### Vaccination intention

At the time when COVID-19 vaccination became available in the Netherlands (January 2021), the vaccination intention increased from around 60% in November 2020 to (77–94%) in January 2021 among all age groups [10]. In round 21 (September 2022), 96% of participants had received at least one vaccination which proved to be higher than the registered vaccination rate in the Dutch population (84%) [14]. Three important reasons for people to be willing to be vaccinated (in January 2021) were to protect oneself, to protect loved ones, and a moral obligation to contribute to the collective end to the crisis [15]. Participants who were initially hesitant about vaccination but switched to intention to get vaccinated, were less uncertain about short- and long-term effects of the vaccination, were more likely to trust the government, and were more likely to believe that the vaccine protects others and is safe [10]. Another important factor was whether participants had received an invitation to get vaccinated. Participants who did not get the vaccine also had various motivations, such as feeling pressured by the government to get vaccinated, having concerns about long-term side effects of vaccination, or having confidence in their own immune system [15].

### Policy support and trust in the government

COVID-19 preventive measures have been introduced to protect public health, but their adverse psychological, social, and economic impact may have weakened their popular support over time. We found that during the first two years of the pandemic, overall support declined for all measures and was systematically lower for those measures that were more socially restrictive [16]. More specifically, differences in support were most evident among different age groups (i.e., higher support for hygienic measures among younger participants and higher support for measures restricting social contacts among older participants), and less so for other demographical factors such as living situation or educational level. Also, patterns of support did not seem to reflect patterns in numbers of COVID-19 hospital admissions and strictness of the COVID-19 preventive measures.

### Well-being

We found that younger participants (under 40s and even more so with the under 26 age group) reported increasingly higher levels of loneliness throughout the pandemic compared to older participants, particularly in times when COVID-19 preventive measures were stricter [17]. Younger participants were also more likely to report both positive (eg, working from home) and negative effects (eg, impact on social life) [18]. Participants stated that these negative effects were mostly the result of imposed measures.

### Further reading

The Corona Behavioral Unit has published its survey findings (usually within two weeks after data collection) on the website of the National Institute for Public Health and The Environment (RIVM), as commissioned by the Dutch Ministry of Health, Welfare and Sport, see rivm.nl/gedragsonderzoek/maatregelen-welbevinden (in Dutch, with English summaries), and rivm.nl/en/coronavirus-covid-19/research/behaviour.

## Strengths and limitations

The cohort has several strengths. It is a large, nation-wide, dynamic on-going cohort with more than 189,000 participants that have contributed to more than 1,000,000 records (between 35,000–90,000 responses every data collection round) during more than two years into the COVID-19 pandemic, with an attrition rate of 31% (i.e., individuals who had at least one completed questionnaire during the last four rounds). The cohort has provided extensive questionnaire data on self-reported adherence to COVID-19 preventive measures, support for and determinants of those measures, trust in the Dutch government, well-being, use of media. These insights have informed nation-wide governmental policies (eg, introduction of a curfew, coronavirus entry pass), that might have otherwise relied on assumptions about people’s well-being and behavior. The questionnaire was informed by a theoretical model, comprised of the Health Belief Model and five key constructs relevant for the maintenance of behavior change [12]. In addition, the study was designed following mixed methodology with the use of open-ended questions, in-depth interviews and focus groups amongst cohort participants. This mixed-methods design enables us to complement and add insights to the quantitative data, and to improve the quality of the questionnaire. The cohort also has a high turnover of questionnaires, capturing changes in constructs within persons over time–as the context (policies, transmission rates) keep changing during the pandemic.

Several weaknesses of the cohort should also be mentioned. Participants are recruited via existing Public Health Service (GGD) panels (i.e., people who already participated in health research), municipal panels, via social media (eg, Facebook), and via mailing lists of higher education organizations. Furthermore, our questionnaire is only available online and for those who master the Dutch language. With respect to the interviews, only those who first fill in the questionnaire and subsequently answer their phone are interviewed. All these approaches are sensitive to selection bias, so that results from this cohort should not be generalized to the general Dutch population. However, the cohort results are compared to those from a 3-weekly, cross-sectional trend study in a demographically representative sample of the Dutch population completing partly the same questionnaire [19]. Cross-sectional results (eg, adherence to social distancing) typically differ by 5 to 10 percentage point. Also, the cohort solely relies on self-reported behavior, which may differ from actual behavior due to social desirability, underreporting of unconscious rule violations, or forgetfulness. Another limitation is that participants are randomized to sub-cohorts based on a specific top (eg, well-being) to reduce participant burden, however complicating analyses across these sub-cohorts. Finally, because the Corona Behavioral Unit is established during the pandemic there are no available baseline (pre-pandemic) measurements for non-COVID-19-specific outcomes such as well-being. As such, we cannot control for any potential pre-existing differences in outcomes, nor can we make a baseline comparison for those outcome measures.

## Conclusion

In response to the COVID-19 pandemic, the Corona Behavioral Unit cohort was established at the start of the pandemic in April 2020 by the Dutch National Institute for Public Health and the Environment (RIVM) and the 25 regional public health services in the Netherlands. Over a two-and-a-half year period, the cohort provided detailed insights in trends over time about COVID-19 preventive behaviors of Dutch citizens, what they thought of the imposed measures and how they were doing physically, mentally and socially. These insights informed COVID-19 policy making and pandemic communication in the Netherlands during the pandemic by identifying key beliefs and misconceptions underlying people’s behaviors. The cohort data will continuously be used to examine longitudinal between- and within-person associations on demographics, health, social and adherence behaviors with key themes such as psychosocial variables, trust and well-being. As we have been able to run this cohort during more than two and a half years of the COVID-19 pandemic, we are also planning to include in the analyses the effects of contextual factors such as the imposed COVID-19 preventive measures and the Dutch government’s COVID-19 press conferences that were used to inform the public. These data will not only add to our understanding of people’s perceptions, behaviors and well-being during the COVID-19 pandemic for future pandemic preparedness, they also provide a unique opportunity to study how beliefs, affect, and behaviors evolved in the context of health behaviors that are completely new to people.

## Supporting information

S1 Text Informed consent as used in the Corona Behavioral Unit cohort study. (DOCX)

S1 Table Responses of the Corona Behavioral Unit cohort study, April 2020 –September 2022. (DOCX)

S2 Table Number of different open-ended items that have been embedded in the survey of the Corona Behavioral Unit cohort study, April 2020 –September 2022. (DOCX)
