# Supplementary material for: Cohort profile: The Corona Behavioral Unit cohort, a longitudinal mixed-methods study on COVID-19-related behavior, well-being and policy support in the Netherlands
Source: PLoS One. 2023 Jul 31;18(7):e0289294. doi: 10.1371/journal.pone.0289294 (PMC10389736; doi:10.1371/journal.pone.0289294)
Supplement: S1 Text — (DOCX) [file pone.0289294.s001.docx]

**S1 Text. Informed consent as used in the Corona Behavioral Unit cohort study.**

**Informed consent**
If you agree to participate and complete the questionnaire, you consent to us processing your data for this study, according to the following conditions:

- Your participation in this study is completely voluntary and you can withdraw from the study and withdraw your consent at any time without giving reasons. Your rights will not be affected by the decision to stop the study.
- Your data will only be processed for the purpose of the study and in accordance with the applicable privacy statement.
- Your personal data will be treated confidentially. We do not know who gave which answers because the answers are separated from your email address and telephone number.
- Your personal data is appropriately secured. Your data can only be viewed by a few employees of the RIVM, Research2Evolve and the GGD with a duty of confidentiality. The data is kept securely so that unauthorized persons cannot access it.

The data will be kept for a minimum of 10 years after completion of the study.
