# Supplementary material for: Cohort profile: The Corona Behavioral Unit cohort, a longitudinal mixed-methods study on COVID-19-related behavior, well-being and policy support in the Netherlands
Source: PLoS One. 2023 Jul 31;18(7):e0289294. doi: 10.1371/journal.pone.0289294 (PMC10389736; doi:10.1371/journal.pone.0289294)
Supplement: S1 Table — (DOCX) [file pone.0289294.s002.docx]

**S1 Table. Responses of the Corona Behavioral Unit cohort study, April 2020 – September 2022.**

|  |  |  |  |  |  |  | Completed | | |  | Not completed | | | |
| --- | --- | --- | --- | --- | --- | --- | --- | --- | --- | --- | --- | --- | --- | --- |
| Round |  | Study period |  | Unique participants | Response rate^1^ | Invited (FU) or started (B) | Total | Matched^2^ | Un-matched |  | Total | Other^3^ | Not started | Not finished |
| 1 | 2020 | 17-24 Apr | B | 89943 |  | 89943 | 89943 |  |  |  |  |  |  |  |
| 2 |  | 7-12 May | FU | 89943 | 82% | 65764 | 53659 | 53200 | 459 |  | 12105 | 1080 | 9468 | 1557 |
| 3 |  | 26 May-1 Jun | FU | 106465 | 75% | 64397 | 48213 | 47834 | 379 |  | 16184 | 217 | 13767 | 2200 |
|  |  |  | B |  |  | 18985 | 16522 |  |  |  | 2463 | 2463 |  |  |
| 4 |  | 17-21 Jun | FU | 106465 | 70% | 73218 | 51208 | 50828 | 380 |  | 22010 | 269 | 19354 | 2387 |
| 5 |  | 8-12 Jul | FU | 111948 | 64% | 72181 | 45975 | 45610 | 365 |  | 26206 | 165 | 23699 | 2342 |
|  |  |  | B |  |  | 6930 | 5483 |  |  |  | 1447 | 1368 |  | 79 |
| 6 |  | 19 -23 Aug | FU | 129133 | 63% | 73048 | 45817 | 45451 | 366 |  | 27231 | 233 | 24113 | 2885 |
|  |  |  | B |  |  | 18816 | 17185 |  |  |  | 1631 | 1292 |  | 339 |
| 7 |  | 30 Sep-4 Oct | FU | 129133 | 62% | 80451 | 49574 | 49115 | 459 |  | 30877 | 186 | 27884 | 2807 |
| 8 |  | 11-15 Nov | FU | 148758 | 59% | 79235 | 46690 | 46228 | 462 |  | 32545 | 208 | 29388 | 2949 |
|  |  |  | B |  |  | 22093 | 19625 |  |  |  | 2468 | 1979 |  | 489 |
| 9 | 2021 | 30 Dec-3 Jan | FU | 148758 | 62% | 87341 | 54290 | 53751 | 539 |  | 33051 | 170 | 30173 | 2708 |
| 10 |  | 10-14 Feb | FU | 157380 | 56% | 86366 | 48686 | 48218 | 468 |  | 37680 | 235 | 34395 | 3050 |
|  |  |  | B |  |  | 9730 | 8622 |  |  |  | 1108 | 1108 |  |  |
| 11 |  | 24-28 Mar | FU | 157380 | 57% | 89373 | 50636 | 50120 | 516 |  | 38737 | 244 | 35358 | 3135 |
| 12 |  | 5-9 May | FU | 168663 | 54% | 88189 | 47735 | 47276 | 459 |  | 40454 | 188 | 37598 | 2668 |
|  |  |  | B |  |  | 12139 | 11283 |  |  |  | 856 | 646 |  | 210 |
| 13 |  | 16-20 Jun | FU | 175341 | 50% | 93199 | 46151 | 45696 | 455 |  | 47048 | 219 | 43174 | 3655 |
|  |  |  | B |  |  | 7269 | 6678 |  |  |  | 591 | 471 |  | 120 |
| 14 |  | 28 Jul-1 Aug | FU | 175341 | 44% | 94720 | 41324 | 40909 | 415 |  | 53396 | 207 | 50056 | 3133 |
| 15 |  | 8-12 Sep | FU | 183027 | 40% | 93279 | 37081 | 36680 | 401 |  | 56198 | 229 | 51931 | 4038 |
|  |  |  | B | 183027 |  | 8244 | 7686 |  |  |  | 558 | 210 |  | 348 |
| 16 |  | 20-24 Oct | FU | 187737 | 41% | 95244 | 38846 | 38403 | 443 |  | 56398 | 181 | 52766 | 3451 |
| 17 |  | 24-28 Nov | FU |  | 45% | 94093 | 42213 | 41731 | 482 |  | 51880 | 193 | 46677 | 5010 |
|  |  |  | B | 187737 |  | 4801 | 4710 |  |  |  | 91 | 91 |  |  |
| 18 | 2022 | 19-23 Jan | FU | 189619 | 47% | 95295 | 44718 | 44227 | 491 |  | 50577 | 217 | 46859 | 3501 |
| 19 |  | 9-13 Mar | FU |  | 38% | 94281 | 36016 | 35621 | 395 |  | 58265 | 210 | 54271 | 3784 |
|  |  |  | B | 189619 |  | 2059 | 1882 |  |  |  | 177 | 128 |  | 49 |
| 20 |  | 8-12 Jun | FU |  | 35% | 93657 | 33221 | 32838 | 383 |  | 60436 | 180 | 57382 | 2874 |
| 21 |  | 7-11 Sep | FU | 189619 | 37% | 92852 | 34671 | 34283 | 388 |  | 58181 | 168 | 55480 | 2533 |
|  |  | **Total** |  | **189619** | **53%** | **1907192** | **1086343** | **888019** | **8705** |  | **820849** | **14755** | **743793** | **62301** |

B=Baseline questionnaires (new recruits).

FU=Follow-up questionnaires.

^1^ Response rate: Completed follow ups / Invited follow up participants.

^2^ Participant id number, age and sex from the baseline questionnaires were used to match participants on their follow-up questionnaires.

^3^ Other reasons for uncompleted questionnaires are: open link had been used more than once, email address was not correct, survey was closed because participant’s age was below the minimum, participant did not give permission to use their data.
