# Supplementary material for: Cohort profile: The Corona Behavioral Unit cohort, a longitudinal mixed-methods study on COVID-19-related behavior, well-being and policy support in the Netherlands
Source: PLoS One. 2023 Jul 31;18(7):e0289294. doi: 10.1371/journal.pone.0289294 (PMC10389736; doi:10.1371/journal.pone.0289294)
Supplement: S2 Table — (DOCX) [file pone.0289294.s003.docx]

**S2 Table. Number of different open-ended items that have been embedded in the survey of the Corona Behavioral Unit cohort study, April 2020 – September 2022.**

| **Theme** | **Example item** | **Number of different items** |
| --- | --- | --- |
| **Testing** | Reason not to be tested for the coronavirus after contact with an infected person. | 20 |
| **Vaccination** | Once a vaccine against the coronavirus is developed, will you want to be vaccinated? | 13 |
| **Quarantine and isolation** | Despite having a positive corona virus test result I went outside, because… | 8 |
| **Well-being** | What positive effects do you experience in your life as a result of the situation we are in due to the coronavirus? | 4 |
| **Other** | Information about the coronavirus - Which source was important for you in the past 7 days? | 4 |
| **Holidays** | How did you celebrate Christmas? | 3 |
| **Corona pass** | Have you been to a place where the corona pass is mandatory? | 2 |
| **Comments about corona measures** | If you have any comments about the corona measures in general, you can write them below. | 1 |
| **Comments about the survey** | If you have any comments about the questionnaire, please write them below. | 1 |
